# AI-generated vs. human therapist feedback in internet-based CBT for social anxiety disorder: A six-arm randomized controlled trial with deception design

**DOI:** 10.1016/j.invent.2026.101012

**Published:** 2026-10-16

**Authors:** Jón Ingi Hlynsson, Gerhard Andersson, Per Carlbring

**Affiliations:** a Department of Psychology, Stockholm University, Stockholm, Sweden; b Department of Behavioural Sciences and Learning, Linköping University, Linköping, Sweden; c Department of Health, Education and Technology, Luleå University of Technology, Luleå, Sweden; d Lusófona University, Digital Human-Environment Interaction Labs, Lisboa, Portugal; e University College, Korea University, Seoul, 02481, South Korea

**Keywords:** Social anxiety disorder, Internet-delivered cognitive behavioral therapy (ICBT), Artificial intelligence (AI), Therapist feedback, Treatment expectations, Therapeutic alliance, Randomized controlled trial

## Abstract

**Introduction:**

Guided internet-delivered cognitive behavioral therapy (ICBT) is effective for social anxiety disorder (SAD), but therapist-delivered feedback limits its scalability. Whether AI-generated feedback can match human feedback, and whether patients' expectations about the source shape outcomes is unknown.

**Method:**

In a six-arm randomized controlled trial, 263 adults with SAD received psychologist-reviewed AI-generated or human feedback. They were told it came from a human or an AI which was correct or incorrect information (four fully crossed feedback arms), or were allocated to unguided self-help or a waitlist. The Social Phobia Inventory (SPIN) was analyzed using linear mixed models, complemented by Bayesian estimation. Secondary outcomes included therapeutic alliance, treatment credibility, negative effects, depression, and anxiety.

**Results:**

Active treatment yielded large reductions in social anxiety relative to waitlist (*d* = −1.23). Before treatment, participants rated it as more credible when they expected feedback from a human rather than AI. AI and human feedback did not however statistically differ in symptom reduction (*d* = 0.10, 95% CI [−3.62, 5.91]); non-inferiority was not established. Neither the actual source, participants' beliefs, nor their congruence influenced symptoms or alliance. Depression, anxiety, and negative effects did not differ by source. Stronger alliance predicted subsequent symptom reduction, but not the reverse.

**Conclusions:**

Psychologist-reviewed AI-generated feedback yielded reductions in social anxiety symptoms that did not differ significantly from human feedback, without greater harm, and despite participants' initial expectation that AI would be less credible. The barrier to AI-supported ICBT may therefore lie in expectations rather than outcomes.

**Trial registration:**

ISRCTN11343758.

## Highlights

• 263 adults with social anxiety received psychologist-reviewed AI or human feedback
• The AI vs human symptom reduction difference was not statistically significant
• Non-inferiority of AI-generated feedback was not established
• No significant source, belief, or interaction effects on symptoms or alliance
• Stronger alliance predicted later symptom reduction, but not the reverse

## 1 Introduction

Since the late 1990s, Internet-based psychological interventions have seen a significant increase in adoption and innovation (Zale et al., 2021). Designed to parallel conventional face-to-face therapy in length and content (Vernmark et al., 2024), these interventions range from live, real-time video therapy sessions, such as telepsychiatry via platforms like Zoom, to self-guided digital self-help programs, with or without asynchronous human guidance (Andersson, 2025). Importantly, there is much empirical support for the efficacy of Internet-based psychotherapy. For instance, guided Internet-based Cognitive Behavioral Therapy (ICBT) was found to be as effective as traditional face-to-face therapy for a wide range of psychiatric and somatic disorders in a recent meta-analysis (Hedman-Lagerlöf et al., 2023). However, human guidance is slightly more efficacious than technological guidance, both for symptom reduction and for adherence (Koelen et al., 2022). Although human guidance is clearly advantageous, expanding such personalized treatment to satisfy global needs presents a challenge (Smith et al., 2023).

The integration of Artificial Intelligence (AI) into ICBT presents a potential solution to this scalability issue. Advances in AI technology, particularly large language models (LLMs) and generative AI, offer the potential to automate aspects of therapeutic feedback, potentially mirroring the therapeutic guidance provided by human therapists (Stade et al., 2024). This could take the form of virtual psychoeducational coaches available around the clock or AI entities capable of delivering sophisticated, clinically informed feedback, such as Socratic questioning to aid clients in analyzing negative thought patterns or disputing maladaptive core beliefs (Carlbring et al., 2023).

Recent studies have highlighted the potential for human-AI collaboration in enhancing mental health support, indicating that partnerships between humans and AI could foster as empathic interactions as those facilitated solely by humans. This was exemplified in a randomized controlled trial where human-AI collaboration in text-based peer-to-peer mental health support resulted in conversations deemed 19.6% more empathic (Sharma et al., 2023). More broadly, clinicians and laypeople often classify therapeutic responses near chance and sometimes prefer the AI in blinded tests (Franke Föyen et al., 2025; Hatch et al., 2025; Pareek and Jain, 2025). Experts have rated LLM-delivered behavioral activation at or above human trainees (Napiwotzki et al., 2025), and two fully AI-delivered smartphone treatments for SAD recently yielded moderate reductions in symptom severity (Hlynsson et al., 2026). Finally, AI support has been suggested to raise engagement and improvement in self-guided ICBT for depression (So et al., 2026) and alongside clinician-led care (Habicht et al., 2025). Still, no study to our knowledge has combined a randomized design with a structured ICBT protocol, validated clinical outcome measures, and systematic control for participant expectations about the feedback source.

Matching human feedback on outcomes does not mean that AI feedback engages the same relational processes. The therapeutic alliance, defined as the affective bond and agreement on tasks and goals between therapist and client (Horvath and Greenberg, 1989; Tracey and Kokotovic, 1989), is a commonly reported predictor of psychotherapy outcome. Within-patient improvement in alliance predicts symptom reduction at the following session after accounting for reverse causation (Falkenström et al., 2013, Falkenström et al., 2016). The association is reciprocal (Marker et al., 2013; Xu and Tracey, 2015), and its magnitude increases with symptom severity and treatment length (Zilcha-Mano and Errázuriz, 2015). Whether these findings extend to digital care remains uncertain. Whether a meaningful alliance can be established with an AI agent is still an open question (Malouin-Lachance et al., 2025). Clients do form measurable alliance-like perceptions toward automated systems (Tong et al., 2025). In a cancer-support app with a rule-based conversational agent, higher task and goal alliance with the app was associated with lower later distress (Schläpfer et al., 2026). Finally, alliance ratings predict later symptom reduction in ICBT for SAD (i.e., increased alliance is associated with reduced social anxiety in the following week; Shalom et al., 2025). This raises the question of whether alliance influences the effect of feedback source, and whether that link is causal.

Expectations about the source of support may confound the comparison. If patients who are told their feedback is AI-generated both expect less and improve less, any apparent inferiority would partly reflect expectancy rather than actual feedback. This concern is not trivial in SAD. Patients who received escitalopram but were told it was an active placebo showed less than a third of the response rate of those given the same drug openly (Faria et al., 2017). Credibility and expectancy predict clinical response and treatment completion in both psychotherapy and internet interventions (Dear, 2025; Kumpasoğlu et al., 2025), and therapeutic dialogue is evaluated differently depending on whether an AI or a human is believed to have produced it (Pareek and Jain, 2025). Laypeople blind to the source rated ChatGPT-generated responses to everyday mental health problems as at least as authentic, professional, and practical as human-written responses (Lopes et al., 2023); when the source was disclosed, ratings of human responses increased while ratings of AI responses were largely unchanged (Jain et al., 2024).

### 1.1 Aims and objectives

It is estimated that up to 40% of individuals suffering from SAD do not achieve remission following state-of-the-art treatments (Leichsenring and Leweke, 2017). Despite ICBT's greater scalability over conventional methods (Andersson et al., 2019), its scalability is limited by the necessity for therapists to devote approximately 20 min per week to provide individualized feedback to each patient (Brunner et al., 2026). In the present study, AI was integrated to automate the feedback process, aiming to enhance the therapy's scalability and efficiency. To separate actual from perceived source, some participants were told one source but received the other, and all were offered full disclosure at the end of treatment; the Swedish Ethical Review Authority approved this procedure. Because feedback may confirm or challenge source-based expectations, congruence between actual and believed source could also matter for symptoms and alliance, which is why we examine their interaction. Combined with a transparent-condition arm, a self-help control, and a waitlist control, this design yields a six-arm trial that estimates the effects of receiving feedback, of feedback source, of believed source, and their interactions. To that end, the following research questions were put forth.

### 1.2 Research questions

1) Is AI-generated feedback non-inferior to human therapist feedback on social anxiety symptoms (SPIN total score) at post-treatment, within a pre-specified non-inferiority margin of 0.30 SD?
2) Independent of the actual feedback source, does the believed source of feedback (told AI vs. told human) affect symptom trajectory and therapeutic alliance, and do actual and believed source interact?
3) Exploratory: Among feedback recipients, how do therapeutic alliance and social-anxiety symptoms relate to one another over the course of treatment, as estimated by a cross-lagged panel model?
4) Does feedback-guided ICBT outperform unguided self-help, and does active treatment outperform the waitlist control?

## 2 Method

### 2.1 Participants and design

The study enrolled adult participants who met inclusion criteria for Social Anxiety Disorder (SAD) based on a cutoff score on the Liebowitz Social Anxiety Scale self-report (LSAS-SR) (Liebowitz, 1987; dos Santos et al., 2013), aligning with clinical guidelines for assessment (National Institute for Health and Care Excellence (NICE), 2013). Recruitment was carried out using targeted social media and widespread advertising to broaden demographic reach (Lindner et al., 2015). Recruitment was conducted through a dedicated online platform (Vlaescu et al., 2026).

Interested individuals initially completed self-report questionnaires to assess their preliminary eligibility. Those who met these initial criteria advanced to complete the LSAS-SR. Participants were required to score ≥ 30 on the LSAS-SR (Rytwinski et al., 2009) and meet none of the exclusion criteria, which included significant risk of suicide, suicide attempts in the last three months, severe major depression (scores ≥20 on the Patient Health Questionnaire 9-item) (Kroenke et al., 2001), ongoing participation in other psychological treatments, unstable psychotropic medication in the last month, and primary diagnoses other than SAD.

After providing informed consent, participants were randomized into one of six groups, using permuted blocks of unknown size automated through the Iterapi platform, to evaluate how the feedback type and their awareness of its source influence treatment outcomes. The first two groups were informed accurately whether they were receiving AI-generated or human feedback. The next two groups were misled regarding the feedback source: one led to believe that AI-generated feedback came from humans, and the other that human feedback was AI-produced. Additionally, one group participated in the ICBT program without any feedback to assess the treatment's standalone effectiveness. A waitlist control group was also included, receiving the full program after the nine-week period, to provide a comparison for change occurring without access to treatment.

### 2.2 Intervention

The treatment intervention was a 9-week therapist guided ICBT program based on a Swedish self-help manual, specifically designed for individuals with SAD (Furmark et al., 2019). Nine modules were delivered over the course of the 9-week intervention, enabling participants to independently work through its content. For instance, participants received psychoeducation, conducted behavioral experiments, and engaged in exposure exercises to confront social fears (a more detailed overview of the treatment content is provided in Table S1). The human-feedback arms were served by 13 therapists (10 psychology students in their final semester of clinical training and three licensed psychologists), with patients unevenly distributed across them. Therapists were aware that the trial included AI-generated feedback conditions and that their own feedback could be presented as either human-written or AI-generated. Because the believed-source framing was randomized within each therapist's caseload, they did not know which framing a given participant had received, and all wrote their feedback in their own words, as a human therapist, in all cases. The unguided self-help condition was provided with the same nine modules but without written feedback, and waitlist participants received no intervention during the 9-week period but were offered the full program afterward.

Participants in the AI conditions received psychologist-reviewed AI-generated feedback. A large language model (LLM) drafted each response, and a licensed psychologist reviewed it before delivery. The model was instructed to match the style and clinical content of human therapist feedback so that its guidance remained therapeutically sound.

#### 2.2.1 Secure and ethical implementation of AI-generated feedback

The study utilized OpenAI models (o4 during the first weeks of the trial, GPT-5.1 thereafter), running on our own dedicated virtual server in Sweden within Microsoft Azure. To further safeguard privacy, no data were stored in Azure. Instead, only specific homework responses from participants were temporarily fed into the model for processing. The model did not store or retain any text, nor was it used for training or improving OpenAI models. Additionally, no personally identifiable or sensitive demographic information, such as age, gender, or other revealing data, was submitted to the model.

Each AI-generated response was carefully monitored and reviewed before being sent to participants. A licensed psychologist assessed every output to ensure accuracy, appropriateness, and therapeutic relevance. In practice, no message was altered. AI-generated feedback followed the same planned schedule as human therapist feedback, with asynchronous written correspondence about once per week after homework submission during the nine-week treatment period. After review, responses were securely transmitted to participants via a separate encrypted internet treatment platform, ensuring full data protection and ethical oversight.

### 2.3 Measures

#### 2.3.1 Liebowitz social anxiety scale self-report (LSAS-SR)

The LSAS-SR (Liebowitz, 1987; dos Santos et al., 2013) is a 24-item scale measuring fear and avoidance of social situations during the past week. The total score is calculated by adding the sum of all fear ratings to the sum of all avoidance ratings. Previous studies have corroborated its diagnostic validity (e.g., Baroni et al., 2023; Rytwinski et al., 2009), indicating that a score of ≥30 on the LSAS-SR is diagnostically predictive of SAD (Rytwinski et al., 2009). The LSAS-SR was administered in the pre-treatment assessment to determine the probable presence of SAD (cf. clinical guidelines for assessment of SAD; NICE, 2013). The LSAS-SR exhibited strong internal reliability (α = 0.95; ω = 0.96).

#### 2.3.2 Social phobia inventory (SPIN)

The Social Phobia Inventory (SPIN) (Connor et al., 2000) is a 17-item self-report scale that indexes fear, avoidance, and physiological discomfort over the past week. Each item is rated on a 5-point scale, ranging from 0 (“not at all”) to 4 (extremely), with total scores between 0 and 68 points. The SPIN has well-established validity (Connor et al., 2000; Osório et al., 2010; Radomsky et al., 2006; for a review, see Mörtberg and Jansson Fröjmark, 2019), and was used as the primary outcome; administered weekly and at post-assessment. The SPIN exhibited strong internal reliability (α = 0.89; ω = 0.92).

#### 2.3.3 Generalized anxiety disorder scale & patient health questionnaire

The Generalized Anxiety Disorder scale (GAD-7), used to assess anxiety symptoms, is a brief 7-item self-report measure that quantifies anxiety symptom severity (Spitzer et al., 2006). The GAD-7 has been found to be sensitive to various anxiety disorders (Kroenke et al., 2010) and has well-established validity in clinical settings and online administrations (Byrd-Bredbenner et al., 2021; Hlynsson and Carlbring, 2024; Martin-Key et al., 2022). The GAD-7 exhibited strong internal reliability (α = 0.86; ω = 0.89).

The 9-item Patient Health Questionnaire (PHQ-9) is a brief self-report measure quantifying depressive symptom severity (Kroenke et al., 2001). Both the full-length and abbreviated 2-item version of the PHQ have demonstrated excellent reliability and validity in clinical settings, among the general population, and in online administrations (Hlynsson et al., 2025; Kroenke et al., 2010; Martin-Key et al., 2022). The PHQ-9 exhibited acceptable-to-strong internal reliability (α = 0.84; ω = 0.88).

The abbreviated GAD-2 and PHQ-2 (Hlynsson and Carlbring, 2024), selected for their effectiveness in monitoring anxiety and depressive symptom severity, were administered on a weekly basis during treatment. The more comprehensive GAD-7 and PHQ-9 were administered during pre-treatment and post-treatment assessment.

#### 2.3.4 Session alliance inventory (SAI)

The Session Alliance Inventory (SAI) (Falkenström et al., 2015b) is a 6-item alliance measure based on the Working Alliance Inventory – Short form Revised (Horvath and Greenberg, 1989). In previous studies, the SAI has demonstrated excellent internal and temporal consistency (Falkenström et al., 2015a; Lindqvist et al., 2023). Participants in the four feedback conditions completed the SAI weekly to assess the therapeutic alliance. The SAI exhibited strong internal reliability, measured at the first session (α = 0.95; ω = 0.97).

#### 2.3.5 Treatment credibility and expectancy questionnaire (CEQ)

The Credibility/Expectancy Questionnaire (CEQ) is a 6-item self-report measure that evaluates the credibility of and expectations toward therapy (Devilly and Borkovec, 2000). The CEQ was administered twice at baseline in counterbalanced-content parallel versions: once with participants instructed to imagine that written feedback on their exercises would be provided by a human therapist, and once imagining that it would be provided by an artificial intelligence. The two versions were identical in item wording apart from the referenced feedback source. Both versions of the CEQ exhibited strong internal reliability (human-referenced: α = 0.87, ω = 0.93; AI-referenced: α = 0.92, ω = 0.97).

#### 2.3.6 Negative effects questionnaire (NEQ)

The Negative Effects Questionnaire (NEQ) is a 20-item self-report measure that evaluates the negative effects of psychological treatment (Rozental et al., 2016, Rozental et al., 2019). Items can be summed to index the frequency of negative effects experienced, categorized by treatment or other circumstances (Rozental et al., 2016, Rozental et al., 2019). Given that the NEQ functions as an index of heterogeneous adverse events rather than a reflective measure of a single latent construct, internal consistency estimates should be interpreted with caution. For transparency, tetrachoric-based reliability of the occurrence items was high at post-assessment (α = 0.88; ω = 0.92).

### 2.4 Ethics and registration

A few words on our use of deception are warranted from an ethical perspective. If participants were fully aware of the true nature of their feedback source, their expectations and biases could markedly influence the outcomes, making it impossible to isolate the actual effects of AI-generated feedback. All participants provided informed consent before enrolling in the study, *explicitly acknowledging* that they may be misled regarding certain aspects of their treatment. Furthermore, to ensure ethical integrity, all participants were offered a full debriefing after the post-treatment assessment, disclosing whether their feedback had come from an AI or a human therapist; approximately 20% wished to know and received the disclosure.

This study has been reviewed and approved by the Swedish Ethical Review Authority on 17 March 2025 (Dnr: 2025–01058-01) and was conducted in accordance with the Declaration of Helsinki. It was retrospectively registered at The UK's Clinical Study Registry (ISRCTN11343758) on 9 April 2026.

### 2.5 Data analysis

The primary outcome (SPIN total score) was analyzed by intention-to-treat using linear mixed-effects models (LMMs). Models included condition, time (categorical: baseline, weeks 1–8, post-treatment), and their interaction as fixed effects, alongside by-participant random intercepts and random slopes (retained because they improved model fit, χ^2^(2) = 215, *p* < .001). The primary comparison tested non-inferiority of AI-generated feedback (pooled *n* = 88) versus human therapist feedback (pooled *n* = 88) evaluated at post-treatment, against a pre-specified 0.30 SD margin on the SPIN (one-sided α = 0.025), which equated to 3.32 SPIN points based on the sample's SD.

Due to early trial cessation, the realized sample (*N* = 263; planned *N* = 480) yielded approximately 50–60% power for the primary non-inferiority test. As such, primary inferences emphasize point estimates and 95% confidence intervals over dichotomous testing. Two sensitivity analyses were conducted: a baseline-adjusted ANCOVA on participants with post-treatment data, and a Bayesian LMM (with weakly informative default priors) reporting the posterior median, 95% credible interval, and region of practical equivalence (ROPE). To isolate actual versus believed feedback effects, a 2 (actual source) × 2 (believed source) × time factorial LMM was fitted separately to symptom severity (SPIN) and weekly alliance (SAI; weeks 1–8). Main effects estimate actual and believed source effects; their interaction estimates actual-belief congruence (the deception manipulation). Given the realized per-cell sample (~44), this contrast was pre-specified as exploratory; non-significance implies non-detection rather than no effect.

Two further pre-specified contrasts evaluated the value of feedback per se: feedback-guided ICBT (the four feedback arms combined, *n* = 176) versus unguided self-help (*n* = 44), and active treatment (all five treatment arms combined, *n* = 220) versus waitlist (*n* = 43), each estimated from a mixed model as the adjusted between-group difference at post-treatment with its 95% confidence interval and standardized effect size. The active-versus-waitlist contrast remained well powered at the realized sample (>90%); the guided-versus-self-help contrast was adequately but not generously powered (~70%). Multiplicity across the two comparator contrasts was controlled with a Holm procedure.

As an exploratory analysis, the temporal interplay of therapeutic alliance and social-anxiety symptoms was examined within the feedback-receiving arms using a cross-lagged panel model fitted to weekly SAI and SPIN assessments, aggregated into three treatment phases (weeks 1–3, 4–6, 7–8) with stationarity constraints on the autoregressive and cross-lagged paths. A random-intercept cross-lagged panel model (RI-CLPM; Baribeau et al., 2022; Hamaker et al., 2015) was attempted but not identifiable at the realized sample size and wave structure; the standard cross-lagged model is therefore reported, with estimates interpreted as exploratory between-person associations.

Feedback-source perception was assessed once at post-treatment as a manipulation check (a 0–100 rating of whether participants believed their feedback came from a human or an AI) and is reported separately. Comorbid depression (PHQ-9) and anxiety (GAD-7) were analyzed by baseline-adjusted analysis of covariance comparing actual feedback sources at post-treatment. Negative effects (NEQ-20) were compared across actual and believed feedback source. Pre-randomization CEQ ratings (human- vs. AI-imagined) were compared with a paired-samples *t*-test, with the standardized mean difference as the effect size. Finally, Cronbach's alpha and McDonald's omega were calculated to evaluate the internal consistency reliability of all measures (Cortina et al., 2020).

All deviations from the registered plan are reported transparently: principally the framing of inference in estimation terms, the retention of a random slope not specified in the registration, and the addition of the exploratory cross-lagged analysis. Analyses were conducted in R (R Core Team, 2021) using lme4 (Bates et al., 2015) and lmerTest (Kuznetsova et al., 2017), emmeans for adjusted contrasts and non-inferiority/equivalence tests (Lenth, 2024), brms (Bürkner, 2017) and bayestestR (Makowski et al., 2019) for the Bayesian analyses, and tidybayes (Kay, 2024) and ggplot2 (Wickham, 2016) for posterior summaries and figures.

## 3 Results

The CONSORT diagram for the study is presented in Fig. 1. In total, 366 potential study participants completed initial screening questionnaires between August 2025 and April 2026; 263 (72%) were eligible and subsequently randomized to one of the six conditions. Descriptive statistics and demographic data are summarized in Table 1. The six arms were comparable at baseline on age, gender, education, and symptom severity, with baseline SPIN means near 40 across conditions. The assumed data-generating process underlying the primary analyses is visualized in **Fig. S1** (cf. Rohrer, 2018).

**Fig. 1:**
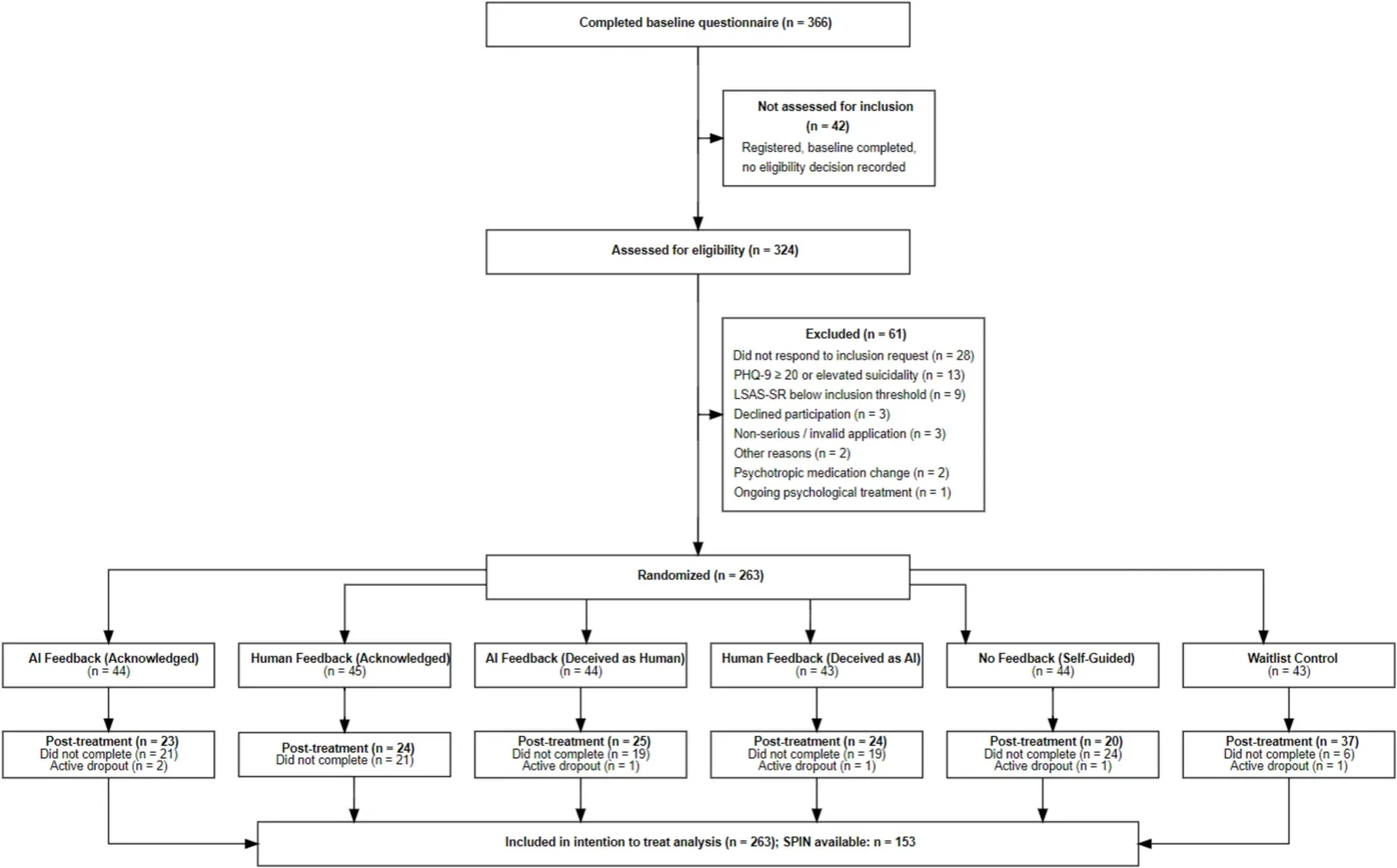
CONSORT diagram.

**Table 1:** Demographic information by group.

Characteristic	Overall (*n* = 263)	AI / Aware (*n* = 44)	Human / Aware (*n* = 45)	AI / Deceived (*n* = 44)	Human / Deceived (*n* = 43)	Self-guided (*n* = 44)	Waitlist (*n* = 43)
Age in years	41.0 (12.1) [18.0–83.0]	37.8 (12.1) [18.0–65.0]	42.4 (11.2) [18.0–71.0]	39.3 (9.8) [19.0–66.0]	40.5 (12.3) [21.0–70.0]	42.3 (13.4) [20.0–83.0]	43.9 (12.9) [21.0–74.0]
Gender							
Female	203 (77%)	37 (84%)	35 (78%)	33 (75%)	30 (70%)	36 (82%)	32 (74%)
Male	55 (21%)	5 (11%)	8 (18%)	10 (23%)	13 (30%)	8 (18%)	11 (26%)
Other/unsure/declined	5 (1.9%)	2 (4.5%)	2 (4.4%)	1 (2.3%)	0 (0%)	0 (0%)	0 (0%)
Education level							
Primary school	7 (2.7%)	2 (4.5%)	2 (4.4%)	1 (2.3%)	2 (4.7%)	0 (0%)	0 (0%)
High school	57 (22%)	14 (32%)	8 (18%)	9 (20%)	10 (23%)	7 (16%)	9 (21%)
Folk school / vocational	35 (13%)	6 (14%)	7 (16%)	4 (9.1%)	7 (16%)	5 (11%)	6 (14%)
University	158 (60%)	21 (48%)	27 (60%)	29 (66%)	23 (53%)	32 (73%)	26 (60%)
Other	6 (2.3%)	1 (2.3%)	1 (2.2%)	1 (2.3%)	1 (2.3%)	0 (0%)	2 (4.7%)
Occupation							
Working	168 (64%)	27 (61%)	33 (73%)	29 (66%)	25 (58%)	29 (66%)	25 (58%)
Studying	37 (14%)	9 (20%)	6 (13%)	6 (14%)	5 (12%)	5 (11%)	6 (14%)
Parental leave	7 (2.7%)	0 (0%)	0 (0%)	2 (4.5%)	3 (7.0%)	1 (2.3%)	1 (2.3%)
On sick leave	11 (4.2%)	3 (6.8%)	2 (4.4%)	1 (2.3%)	2 (4.7%)	2 (4.5%)	1 (2.3%)
Unemployed	16 (6.1%)	3 (6.8%)	2 (4.4%)	3 (6.8%)	3 (7.0%)	2 (4.5%)	3 (7.0%)
Other	24 (9.1%)	2 (4.5%)	2 (4.4%)	3 (6.8%)	5 (12%)	5 (11%)	7 (16%)
Ever assessed/treated for mental health	171 (65%)	28 (64%)	28 (62%)	28 (64%)	31 (72%)	27 (61%)	29 (67%)
Current psychotropic medication							
Yes, currently	64 (24%)	10 (23%)	8 (18%)	11 (25%)	11 (26%)	12 (27%)	12 (28%)
Yes, previously	15 (5.7%)	2 (4.5%)	3 (6.7%)	2 (4.5%)	3 (7.0%)	2 (4.5%)	3 (7.0%)
No	184 (70%)	32 (73%)	34 (76%)	31 (70%)	29 (67%)	30 (68%)	28 (65%)
Have you ever been evaluated by a psychologist/psychiatrist for ADHD, autism or Asperger's syndrome?	50 (19%)	10 (23%)	7 (16%)	7 (16%)	9 (21%)	8 (18%)	9 (21%)
If you had the freedom to choose, what type of feedback would you have preferred (AI or human)?							
Strong preference for feedback from a human therapist	71 (27%)	14 (32%)	14 (31%)	11 (25%)	12 (28%)	11 (25%)	9 (21%)
Moderate preference for feedback from a human therapist	58 (22%)	8 (18%)	9 (20%)	10 (23%)	9 (21%)	11 (25%)	11 (26%)
No particular preference between human and AI-generated feedback (neutral)	97 (37%)	12 (27%)	19 (42%)	22 (50%)	14 (33%)	15 (34%)	15 (35%)
Moderate preference for AI-generated feedback	21 (8.0%)	4 (9.1%)	1 (2.2%)	1 (2.3%)	6 (14%)	4 (9.1%)	5 (12%)
Strong preference for AI-generated feedback	14 (5.3%)	5 (11%)	1 (2.2%)	0 (0%)	2 (4.7%)	3 (6.8%)	3 (7.0%)
Other	2 (0.8%)	1 (2.3%)	1 (2.2%)	0 (0%)	0 (0%)	0 (0%)	0 (0%)
Do you use AI?	218 (83%)	34 (77%)	38 (84%)	35 (80%)	34 (79%)	42 (95%)	35 (81%)
How often do you use AI?							
Several times a day	76 (35%)	9 (26%)	12 (32%)	11 (31%)	13 (38%)	22 (52%)	9 (26%)
Once a day	19 (8.7%)	2 (5.9%)	4 (11%)	2 (5.7%)	2 (5.9%)	3 (7.1%)	6 (17%)
A few times a week	80 (37%)	13 (38%)	14 (37%)	17 (49%)	11 (32%)	11 (26%)	14 (40%)
Once a week	15 (6.9%)	6 (18%)	3 (7.9%)	1 (2.9%)	1 (2.9%)	2 (4.8%)	2 (5.7%)
Less than once a week	28 (13%)	4 (12%)	5 (13%)	4 (11%)	7 (21%)	4 (9.5%)	4 (11%)
LSAS-SR (baseline)	73.4 (22.5) [27.0–132.0]	76.3 (22.9) [33.0–130.0]	74.2 (24.8) [36.0–132.0]	70.1 (21.9) [27.0–131.0]	74.8 (19.8) [31.0–122.0]	68.6 (20.9) [32.0–110.0]	76.2 (24.1) [31.0–127.0]
SPIN-17 (baseline)	40.5 (11.1) [10.0–68.0]	40.2 (11.0) [19.0–62.0]	40.2 (11.6) [15.0–68.0]	39.7 (10.7) [13.0–66.0]	40.7 (10.2) [10.0–61.0]	39.5 (10.7) [18.0–57.0]	42.7 (12.5) [14.0–62.0]
GAD-7 (baseline)	10.4 (4.9) [0.0–21.0]	9.7 (5.0) [0.0–19.0]	10.0 (4.8) [2.0–19.0]	10.6 (5.2) [1.0–21.0]	10.2 (4.0) [3.0–18.0]	10.4 (4.8) [2.0–20.0]	11.4 (5.6) [0.0–21.0]
PHQ-9 (baseline)	8.8 (5.1) [0.0–24.0]	9.2 (5.6) [0.0–19.0]	8.8 (4.6) [0.0–20.0]	8.3 (5.0) [0.0–19.0]	8.7 (5.3) [1.0–21.0]	8.3 (5.1) [0.0–24.0]	9.6 (5.2) [1.0–21.0]

Note. Data are presented as mean (standard deviation) [Min - Max] or no. (%).

### 3.1 Feedback source and post-treatment social anxiety

The primary analysis tested whether psychologist-reviewed AI-generated feedback was non-inferior to human therapist feedback on post-treatment SPIN scores, using a pre-specified non-inferiority margin of 0.30 SD. The pooled baseline SD was 11.07, giving a margin of 3.32 points on the SPIN. Both feedback sources showed substantial, closely parallel symptom reduction over the course of treatment (see Fig. 2), falling from a baseline of approximately 40 to the high-20s on the SPIN (see Table 2).

**Fig. 2:**
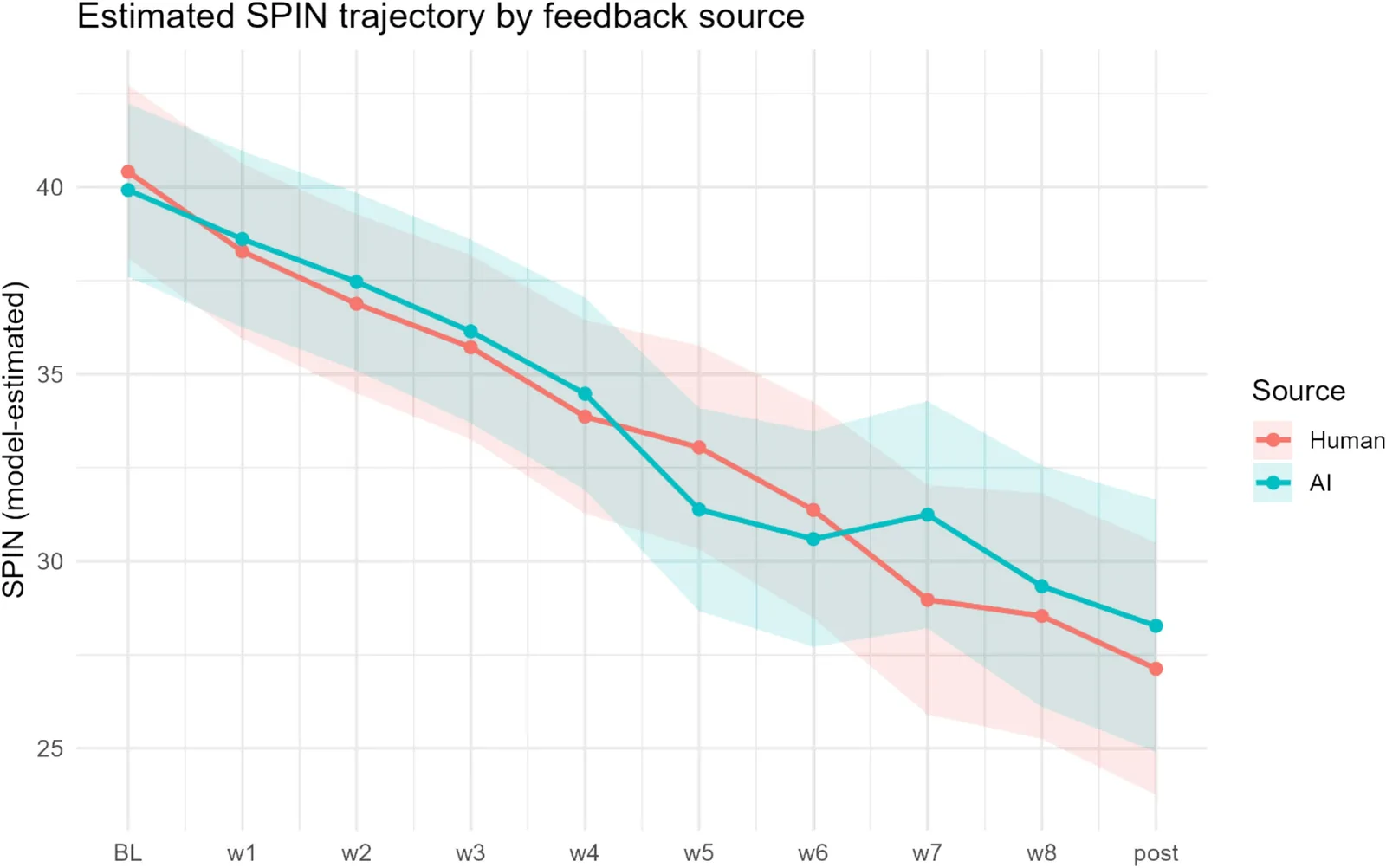
Estimated marginal means of Social Phobia Inventory (SPIN) total scores from the linear mixed model (source × time, with by-participant random intercepts and random slopes) for AI-generated and human-generated feedback across baseline, the eight weekly assessments, and post-treatment. Shaded bands are 95% confidence intervals. Both sources show substantial, closely parallel symptom reduction over treatment; the adjusted between-source difference at post-treatment was 1.15 SPIN points (95% CI [−3.62, 5.91]). Lower scores indicate less social anxiety. BL = baseline; w1–w8 = treatment weeks 1–8; post = post-treatment (week 9).

**Table 2:** Observed SPIN by feedback source, baseline to post-treatment.

Measure	AI	Human
Baseline SPIN, M (SD)	39.9 (10.8)	40.4 (10.8)
Post-treatment SPIN, M (SD)	28.2 (12.4)	24.2 (12.5)
Change, M [95% CI]	−11.6 [−15.0, −8.3]	−14.2 [−17.3, −11.2]
Within-group *d*	−1.08	−1.31
*n* (allocated)	88	88
*n* (post-treatment)	48	48

*Note.* AI and Human columns pool the two AI-source arms (*n* = 88) and the two human-source arms (*n* = 88), respectively; post-treatment *n* reflects participants with a post-treatment SPIN in those arms and are therefore a subset of the 153 analyzed across all six arms.

SPIN = Social Phobia Inventory; higher = worse. Change = post − baseline (negative = improvement), among participants with both assessments. Within-group *d* = mean change / baseline SD (11.07).

At post-treatment, AI feedback was associated with SPIN scores 1.15 points higher than human feedback (SE = 2.41, *df* = 170, 95% CI [−3.62, 5.91]; *d* = 0.10). The point estimate favored human feedback, but the difference was not statistically significant. Because the upper bound of the confidence interval (5.91) exceeded the non-inferiority margin (3.32), non-inferiority was not established (non-inferiority test *p* = .19 (unadjusted)); the two-one-sided-tests equivalence procedure was likewise non-significant (*p* = .19). A baseline-adjusted ANCOVA on participants with post-treatment data (*n* = 96) yielded a directionally similar, though somewhat larger, estimate (AI − human = 3.12, 95% CI [−1.24, 7.48]). Because it conditions on treatment completion, this estimate is susceptible to selection effects from which the mixed model, which uses all available observations, is comparatively protected (cf. Bell et al., 2013); the larger completer estimate should accordingly be interpreted with caution, and the intention-to-treat model retained as primary.

To characterize the difference beyond the dichotomous test, we examined the posterior distribution of the AI − human difference from a Bayesian linear mixed model against a region of practical equivalence of ±0.30 SD (±3.32 points). As Fig. 3 shows, approximately 79% of the posterior fell within this region (median difference 1.07, 95% credible interval [−3.73, 5.75]), with roughly 17% consistent with AI feedback being meaningfully worse and 4% with it being meaningfully better; the corresponding equivalence test was classified as undecided. The primary, sensitivity, and Bayesian analyses are summarized together in Table 3. All three place the AI − human difference near zero, small in magnitude, and inconclusive against the pre-specified margin. Thus, while formal non-inferiority was not demonstrated at the realized sample size, the difference between AI- and human-generated feedback was small, most credible values were clinically negligible, and there was little support for AI feedback being superior.

**Fig. 3:**
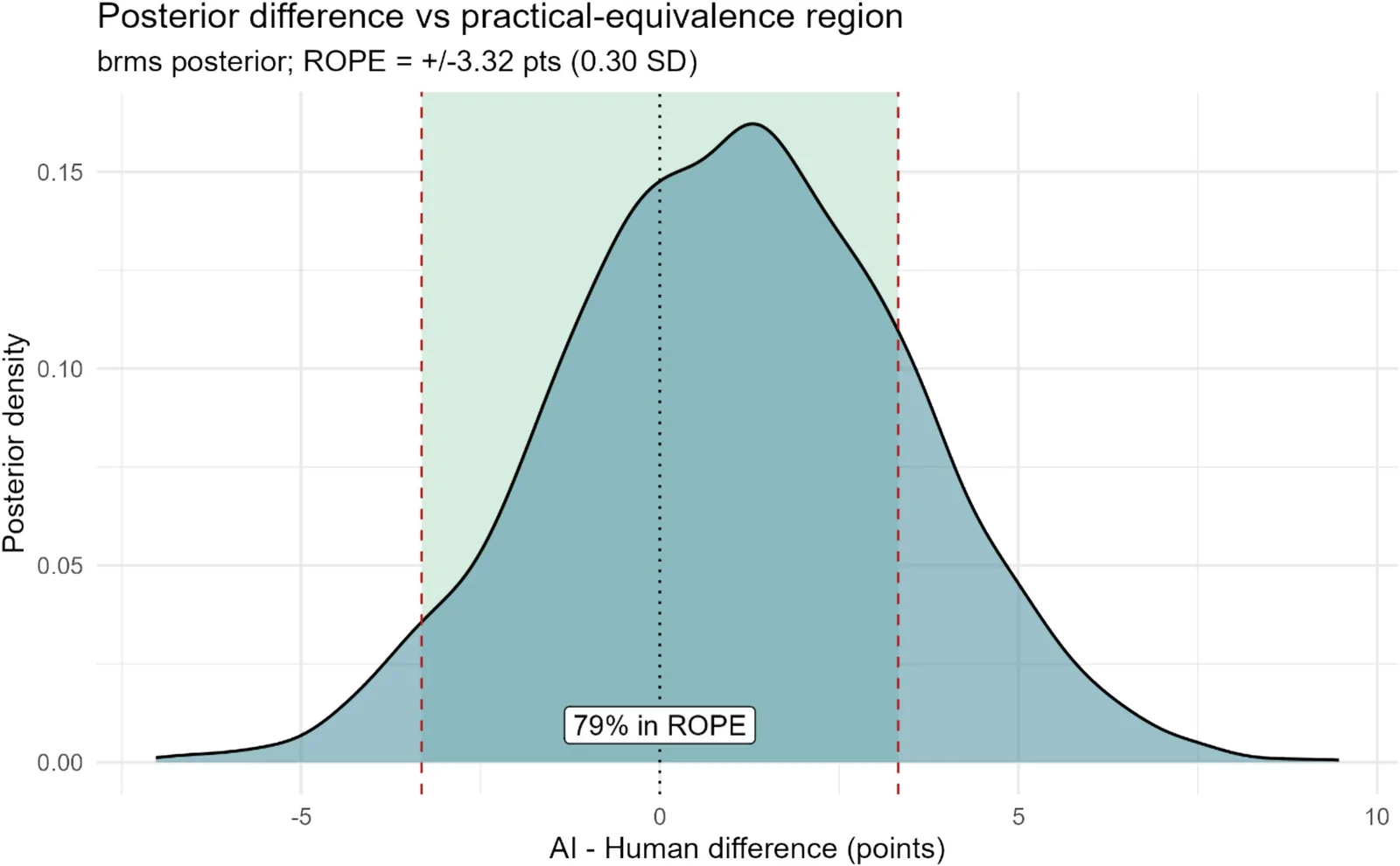
Posterior distribution of the adjusted between-source difference in post-treatment SPIN (AI minus human), from a Bayesian linear mixed model (brms). Positive values indicate higher symptoms (worse outcomes) under AI feedback. The dotted vertical line marks no difference; red dashed lines and the shaded band mark the pre-specified region of practical equivalence (ROPE = ±0.30 SD = ±3.32 SPIN points). Approximately 79% of the posterior falls within the ROPE, ~17% in the region consistent with AI feedback being meaningfully worse, and ~ 4% with it being meaningfully better, indicating a small difference whose most credible values are clinically negligible while formal equivalence remains undecided at the realized sample size.

**Table 3:** AI vs. human feedback on post-treatment SPIN: primary, sensitivity, and Bayesian analyses.

Analysis	AI − Human (SPIN)	95% CI / CrI	*d*	Margin	Conclusion
Primary LMM (ITT, all timepoints; df = 170)	1.15	[−3.62, 5.91]	0.10	3.32	Inconclusive (CI crosses margin)
Sensitivity ANCOVA (completers, *n* = 96)	3.12	[−1.24, 7.48]	0.28	3.32	Inconclusive
Bayesian LMM (ROPE ±0.30 SD)	1.07	[−3.73, 5.75]	–	3.32	79% in ROPE; undecided

*Note.* Positive differences favor human feedback. d = difference / baseline SD. NI margin = 0.30 SD = 3.32 points; non-inferiority concluded if the upper 95% CI bound < margin. CrI = credible interval; ROPE = region of practical equivalence.

These results should be read in estimation rather than decision terms. A non-significant non-inferiority test therefore indicates that comparability could not be established at the pre-specified margin, not that the two feedback sources differ. Interpretation of the source comparison also presupposes assay sensitivity (i.e., that feedback itself constitutes an active ingredient relative to its absence; Mauri and D’Agostino, 2017). Since the guided-versus-unguided contrast reported below (see Table 7) was likewise inconclusive at the realized sample, evidence bearing on non-inferiority should be read jointly with that contrast rather than as standalone support for the efficacy of either feedback source. Both arms improved substantially from baseline; the primary finding is thus substantial symptom reduction under guided ICBT regardless of feedback source, alongside a small and most-credibly negligible difference between sources. The point estimate and the concentration of the posterior within the equivalence region (Fig. 3) are consistent with, but do not confirm, comparable efficacy of AI- and human-generated feedback.

### 3.2 Actual and believed feedback source

The 2 (actual source: AI vs. human) × 2 (believed source: told AI vs. told human) × time factorial mixed model (see Data Analysis) was fitted separately to symptom severity (SPIN) and alliance (SAI). For symptoms, there was a large effect of time (*F*(9, 153.5) = 16.65, *p* < .001): participants improved substantially over treatment regardless of condition (see Fig. 2). Neither the actual source of feedback (*F*(1, 163.8) = 0.04, *p* = .85) nor participants' beliefs about the source (*F*(1, 163.8) = 0.02, *p* = .88) affected symptom trajectories, and the two did not interact (actual × believed *F*(1, 163.8) = 0.02, *p* = .89). At post-treatment, the believed source, actual source, and interaction contrasts were all negligible (see Table 4).

**Table 4:** Actual and believed feedback source: 2 × 2 effects on symptoms and alliance.

Effect	Outcome	Estimate [95% CI]	*F*	*p*
Actual source (AI − human)	SPIN (post)	1.11 [−3.68, 5.90]	0.04	0.853
Believed source (told AI − told human)	SPIN (post)	−0.73 [−5.52, 4.06]	0.02	0.880
Actual × believed (interaction)	SPIN (post)	−0.32 [−9.90, 9.26]	0.02	0.890
Believed source (told AI − told human)	Alliance (SAI)	−0.65 [−2.86, 1.57]	0.33	0.565

Note. Estimates are adjusted between-condition differences at post-treatment (SPIN) or averaged over weeks (alliance). F and p are from Type III joint tests. The actual × believed interaction is pre-specified exploratory.

For therapeutic alliance, ratings increased over the course of treatment (*F*(1, 99.7) = 24.43, *p* < .001; see Fig. 4), but showed the same pattern of null manipulation effects: neither actual source (*F*(1, 142.7) = 1.93, *p* = .17) nor believed source (*F*(1, 142.7) = 0.33, *p* = .56) affected alliance, and they did not interact (*F*(1, 142.7) = 0.10, *p* = .76).

**Fig. 4:**
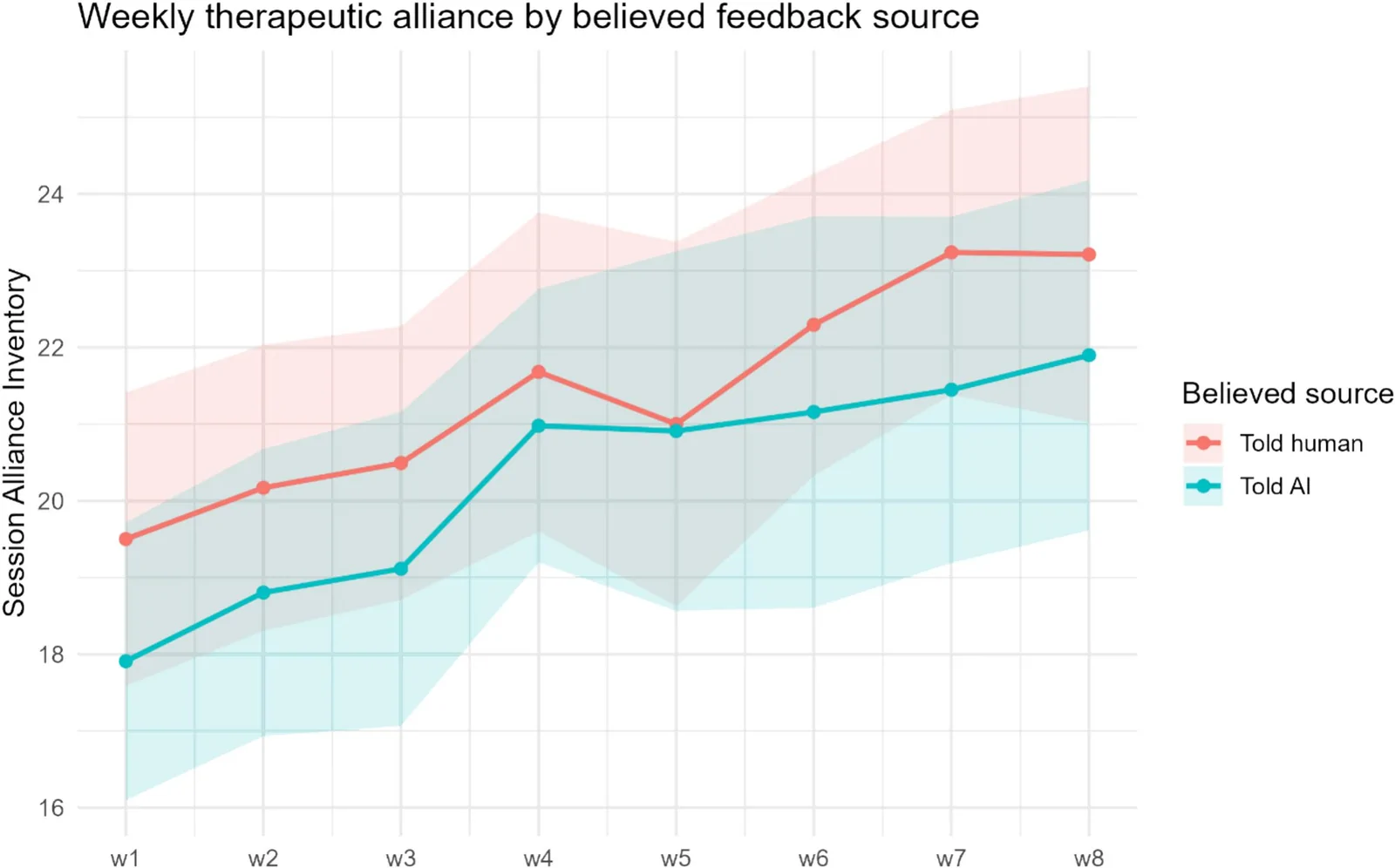
Actual and believed feedback source: 2 × 2 effects on post-treatment symptoms (SPIN) and weekly therapeutic alliance (SAI). Estimates are adjusted between-condition differences at post-treatment (SPIN) or averaged over weeks (alliance); F and p from Type III joint tests. The actual × believed interaction is pre-specified as exploratory.

Taken together, neither the actual source of feedback, participants' beliefs about it, nor the congruence between the two (the deception manipulation) detectably influenced symptom reduction or therapeutic alliance; both improved substantially over treatment across all four feedback conditions (Table 4). Consistent with the ethics-approved protocol we registered, the interaction terms are underpowered at the realized per-cell sample (~44) and are interpreted as non-detection rather than as evidence of no effect.

At post-treatment, participants rated whether they believed their feedback had come from a human or an AI (0 = certain AI, 100 = certain human; Table 5). A 2 × 2 analysis of variance on these ratings confirmed that the belief manipulation registered. Participants told they received human feedback rated the source as substantially more human than those told they received AI feedback (46.5 vs. 26.5; main effect of believed source *F*(1, 88) = 10.81, *p* = .002, partial η^2^ = 0.11, *d* = 0.66). Participants also retained some sensitivity to the true source, rating actual-human feedback as more human than actual-AI feedback independent of what they were told (main effect of actual source *F*(1, 88) = 6.64, *p* = .012, partial η^2^ = 0.07), and the two factors did not interact (*F*(1, 88) = 1.56, *p* = .21). Ratings were nonetheless highly variable (*SD*s ≈ 30) and, apart from the told-human/actual-human condition, tended to fall below the scale midpoint, indicating that the manipulation shifted source perceptions in the intended direction without producing confident conviction. Because this rating was obtained only at post-treatment, it may in part reflect outcome-informed attribution rather than beliefs held during treatment; it should thus read as confirming that the manipulation shifted source perceptions, not as a direct measure of in-treatment belief.

**Table 5:** Post-treatment source perception by actual and believed feedback source.

	*n*	M (SD)
Human / Told human	23	58.3 (31.0)
Human / Told AI	24	30.4 (31.7)
AI / Told human	23	34.8 (33.6)
AI / Told AI	22	22.3 (18.5)

Note. Perception rated 0 (certain AI) to 100 (certain human); 50 = no idea. 2 × 2 ANOVA (sum-to-zero contrasts): believed-source main effect *F*(1, 88) = 10.81, *p* = .002, partial η^2^ = 0.11; actual-source *F*(1, 88) = 6.64, *p* = .012, partial η^2^ = 0.07; interaction *F*(1, 88) = 1.56, *p* = .21.

### 3.3 Temporal dynamics of alliance and symptoms

To examine the temporal interplay of therapeutic alliance and social-anxiety symptoms within the feedback-receiving arms, we fitted a bivariate cross-lagged panel model to alliance (SAI) and symptoms (SPIN) across three treatment phases (weeks 1–3, 4–6, and 7–8, averaged), with autoregressive and cross-lagged paths constrained to equality across intervals (Fig. 5). The standard cross-lagged model is reported (*n* = 86 with complete phase data); associations are between-person and interpreted as exploratory.

**Fig. 5:**
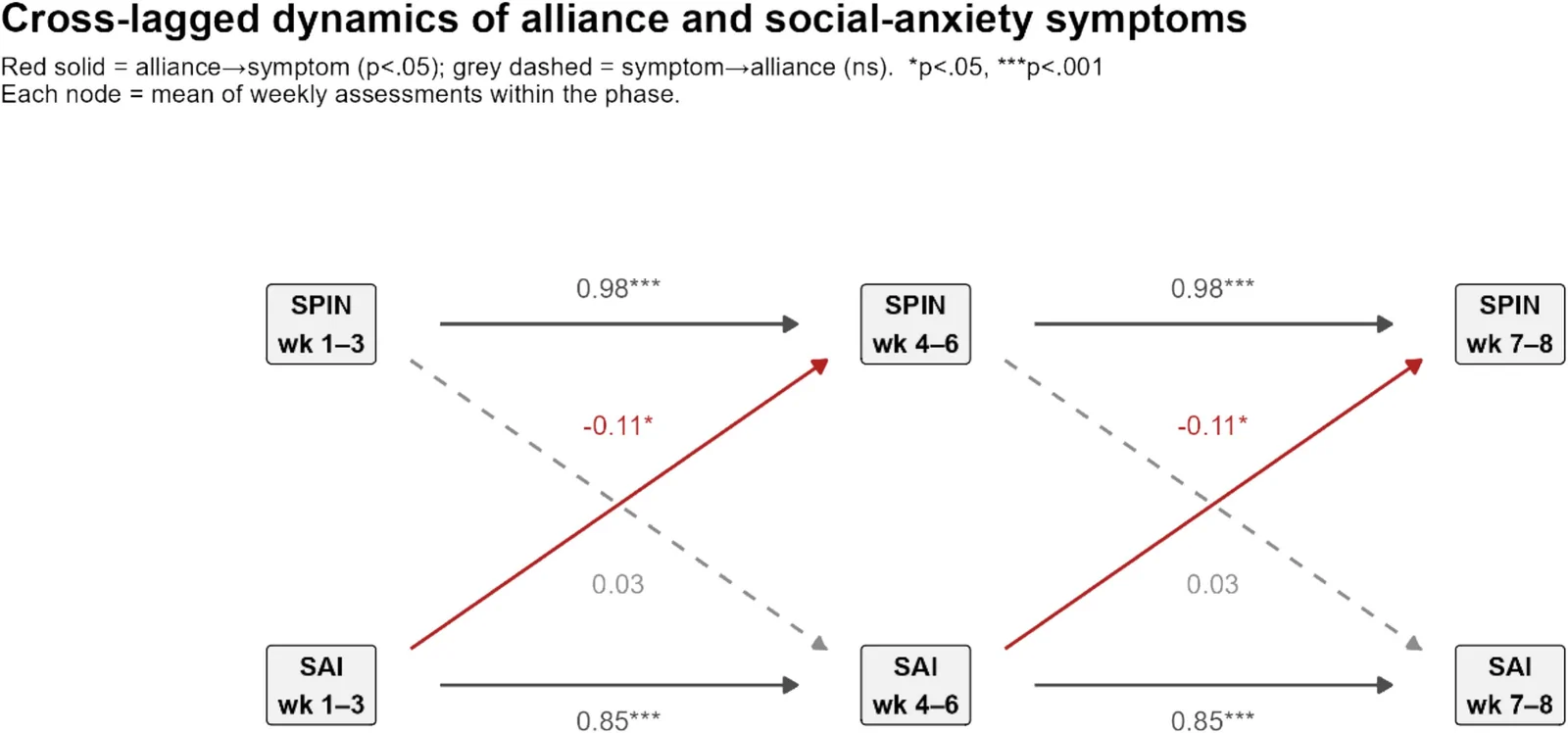
Cross-lagged panel model of therapeutic alliance (SAI) and social-anxiety symptoms (SPIN) across three treatment phases (feedback arms, *n* = 86). Coefficients are unstandardized, constrained equal across intervals. The alliance→symptom cross-lag (red) was significant (b = −0.11, *p* = .037); the symptom→alliance cross-lag (grey, dashed) was not (*p* = .26). Lower SPIN indicates fewer symptoms; a negative alliance→symptom path means higher alliance predicts subsequent symptom reduction. *p < .05, ***p < .001.

The model fit the data well (χ^2^(8) = 14.19, *p* = .08; CFI = 0.99; SRMR = 0.03). Both constructs were highly stable across phases (symptom autoregression = 0.98; alliance autoregression = 0.85, both *p* < .001; Table 6). The cross-lagged paths were asymmetric: stronger therapeutic alliance predicted lower social-anxiety symptoms at the following phase (*b* = −0.11, SE = 0.05, *p* = .037), whereas symptoms did not predict subsequent alliance (*b* = 0.03, *p* = .26). Within the treatment period, alliance thus temporally preceded symptom improvement, but not the reverse (Fig. 5).

**Table 6:** Cross-lagged panel model of alliance and symptoms across treatment phases (feedback arms, n = 86).

Path	b	SE	β (std)	95% CI	*p*
Symptoms → Symptoms (autoregressive)	0.976	0.034	0.904	[0.909, 1.043]	<0.001
Alliance → Symptoms (cross-lag)	−0.112	0.054	−0.068	[−0.218, −0.007]	0.037
Symptoms → Alliance (cross-lag)	0.028	0.025	0.043	[−0.021, 0.077]	0.256
Alliance → Alliance (autoregressive)	0.849	0.040	0.840	[0.771, 0.927]	<0.001

Note. Paths constrained equal across intervals (stationarity). SPIN higher = worse; a negative alliance→symptom coefficient indicates higher alliance predicts lower subsequent symptoms.

Fit: *χ*^*2*^ (8) = 14.19, *p* = .08; CFI = 0.99; SRMR = 0.03. Exploratory.

This directional pattern (i.e., alliance leading symptom change rather than following it) is consistent with therapeutic alliance functioning as an active ingredient rather than a by-product of symptomatic relief. The effect is small. As an exploratory analysis on a modest completer sample, it best read as a informing about direction of associations rather than a confirmed pathway and would not survive correction for multiple comparisons. It nonetheless offers a concrete, testable hypothesis for the completed dataset and for adequately powered replication.

### 3.4 Value of feedback and of active treatment

Feedback-guided ICBT (the four feedback arms combined, *n* = 176) did not differ from unguided self-help (*n* = 44) on post-treatment SPIN (adjusted difference = 0.96, 95% CI [−4.69, 6.60], *d* = 0.09, Holm-adjusted *p* = .74); both improved to similar post-treatment levels (feedback *M* = 26.2, self-help *M* = 24.4). Active treatment (all five treatment arms combined, *n* = 220) substantially outperformed the waitlist control (*n* = 43): participants receiving treatment scored 13.64 SPIN points lower at post-treatment (95% CI [−17.96, −9.31], *d* = −1.23, Holm-adjusted *p* < .001), a large effect, while the waitlist group showed essentially no change (post-treatment *M* = 40.0, versus a sample baseline of approximately 40.5). Both contrasts are reported in Table 7. Table S2 reports recorded message use and module engagement by arm.

**Table 7:** Comparator contrasts on post-treatment SPIN.

Contrast	Difference [95% CI]	*d*	*p* (raw)	*p* (Holm)
Feedback (G1–4) − Self-help (G5)	0.96 [−4.69, 6.60]	0.09	0.739	0.739
Active (G1–5) − Waitlist (G6)	−13.64 [−17.96, −9.31]	−1.23	0.000	0.000

*Note.* Adjusted between-group differences at post-treatment; negative favors the first-named group. d = difference / baseline SD (11.07). Holm correction applied across the two comparator contrasts.

The contrast between guided and unguided treatment should be read in estimation terms. At the realized sample this comparison was only moderately powered (~70%), and self-help retention was limited. Only 20 of 44 allocated participants provided post-treatment data, compared with 96 of 176 in the feedback arms, and thus the comparison rests on a small completer set. The null result therefore indicates that a benefit of personalized feedback over self-help could not be detected at this sample size, not that feedback confers no benefit; the wide confidence interval remains compatible with small-to-moderate differences in either direction. By contrast, the active-versus-waitlist comparison was well powered (>90%), and the large, precisely estimated effect confirms that the intervention produced substantial symptom reduction relative to no treatment.

### 3.5 Secondary outcomes: credibility, negative effects, and comorbid symptoms

#### 3.5.1 Treatment credibility

Participants rated the treatment as substantially more credible when imagining human-delivered feedback than AI-delivered feedback (*M* = 35.0, *SD* = 9.4 vs. *M* = 30.0, *SD* = 11.2), a within-person difference of 5.0 points on the scale (95% CI [4.3, 5.7], *t*(348) = 13.3, *p* < .001; standardized mean difference for the paired comparison = 0.72). A clear majority (74%) rated human feedback as more credible, 14% rated AI feedback as more credible, and 12% rated the two equally. This expectation stands in contrast to the comparable symptom outcomes subsequently observed across actual and believed source (Table 8).

**Table 8:** Secondary outcomes by feedback source.

Outcome	Human	AI	Difference [95% CI]	*p*
Credibility (CEQ)†	35.0 (9.4)	30.0 (11.2)	5.0 [4.3, 5.7]	<0.001
Negative effects (NEQ-20)	3.7 (3.3)	4.4 (3.0)	0.69 [−0.60, 1.98]	0.29
Depression (PHQ-9)	6.0 (4.7)	5.9 (4.8)	0.49 [−0.99, 1.97]	0.51
Anxiety (GAD-7)	5.6 (4.3)	5.7 (4.1)	0.31 [−1.16, 1.78]	0.67

*Note.* Values are M (SD); differences favor human feedback when positive.

†CEQ (Credibility/Expectancy Questionnaire) was rated before randomization by all screened participants before allocation twice, imagining human vs. AI feedback; the difference is within-person (*n* = 349, standardized mean difference = 0.72). NEQ-20 = post-treatment count of negative effects (higher = more), compared by actual source (*n* = 93). PHQ-9 and GAD-7 differences are baseline-adjusted (ANCOVA) between actual sources at post-treatment (*n* = 95).

#### 3.5.2 Negative effects

At post-treatment, participants reported a mean of 4.1 (*SD* = 3.3) negative effects on the NEQ-20. The most commonly endorsed were “I experienced more anxiety” (47.3%), “I experienced more unpleasant feelings” (44.6%), “old, painful memories resurfaced” (43.8%), and “I felt more stressed” (42.9%). This pattern is consistent with the transient distress that commonly accompanies exposure-based treatment. Negative-effect frequency did not differ by actual feedback source (AI *M* = 4.4 vs. human *M* = 3.7; *t*(91) = −1.06, *p* = .29) or by believed source (told-AI *M* = 3.9 vs. told-human *M* = 4.2; *t*(89) = 0.53, *p* = .60); AI feedback was not associated with more negative effects than human feedback. Suicidal ideation (item 10) was endorsed by 11 participants (9.8%); elevated suicidality at screening was an exclusion criterion, and no serious adverse events attributable to the intervention were recorded.

#### 3.5.3 Depression and anxiety

Improvements in comorbid symptoms were comparable across feedback source. Baseline-adjusted post-treatment differences between AI and human feedback were negligible for both depression (PHQ-9: AI − human = 0.49, 95% CI [−0.99, 1.97], *p* = .51) and anxiety (GAD-7: 0.31, 95% CI [−1.16, 1.78], *p* = .67), with both conditions improving from baseline (Table 8). The comparability observed on the primary outcome thus extended to secondary symptom domains.

## 4 Discussion

This trial set out to determine whether psychologist-reviewed AI-generated feedback could substitute human therapist feedback in ICBT for SAD, and whether beliefs about the feedback source shape outcomes. The clearest finding was that active treatment worked; participants receiving any active intervention improved substantially relative to waitlist, a large and well-powered effect, with comparable gains in depression and anxiety. Against this backdrop, the source of feedback mattered remarkably little. AI- and human-generated feedback produced comparable reductions in social anxiety, although non-inferiority was not established at the realized sample. The difference between sources was small, albeit not statistically significant, and most of its posterior fell within the pre-specified region of practical equivalence, which is arguably a more appropriate metric to interpret in this trial than statistical significance (cf. Amrhein et al., 2019). That said, the 95% confidence interval [−3.62, 5.91], extended beyond both −3.32 and + 3.32 SPIN points, so differences larger than the non-inferiority margin in either direction cannot be ruled out. Neither the actual source, participants' beliefs about it, nor the congruence between the two (i.e., the deception manipulation) detectably influenced symptom trajectories or therapeutic alliance, even though a manipulation check confirmed that being told the source shifted participants' perceptions.

The most striking pattern was a dissociation between expectation and outcome. Before beginning treatment, participants rated the identical intervention as substantially more credible when they imagined it delivered by a human than by an AI, a 5-point difference on the credibility scale, with nearly three-quarters preferring human feedback. Yet this pronounced a priori skepticism toward AI did not translate into poorer outcomes, weaker alliance, or more negative effects when participants actually received AI feedback. This contrasts with settings where beliefs about the source strongly shape response; in social anxiety, patients given escitalopram but told it was an active placebo showed roughly a third of the response rate of those told the truth (Faria et al., 2017), whereas here an analogous belief manipulation shifted source perceptions without moving symptoms or alliance. One possibility is that credibility imagined at screening captured general assumptions about AI rather than expectations that operate in treatment. Another possibility is that the manipulation check showed shifted perceptions but limited conviction. Alternatively, structured, asynchronous written feedback makes content more salient than source. Since none of these explanations were tested, we do not draw implementation conclusions from this but put this forth as possible explanations for the discrepancy between expectations and observed outcomes.

The gap between anticipated and realized value has practical implications. Initial reluctance toward AI-supported care may reflect expectations that experience does not confirm, and credibility concerns measured before treatment may be a poor guide to how such interventions actually perform. This is consistent with blinded evaluations in which AI-generated responses are rated on par with human ones until the source is disclosed (Jain et al., 2024; Lopes et al., 2023), and parallels the broader literature on algorithm aversion (see Dietvorst et al., 2015; Longoni et al., 2019; Zhou et al., 2026).

The temporal analysis offered a complementary, exploratory signal about why treatment worked. Within the feedback arms, stronger therapeutic alliance predicted subsequent symptom reduction, whereas symptoms did not predict subsequent alliance. This suggests that alliance may function as a leading contributor to change rather than a by-product of feeling better, a pattern that held regardless of feedback source, offering tentative evidence on the open question of whether a working alliance forms toward AI-supported care (Malouin-Lachance et al., 2025; Schläpfer et al., 2026; Tong et al., 2025). This is also directionally consistent with within-patient alliance–outcome dynamics in psychotherapy (Falkenström et al., 2013, Falkenström et al., 2016) and with early alliance predicting later symptom reduction in ICBT for social anxiety specifically (Shalom et al., 2025).

Negative effects were common but not source-dependent. Many participants reported at least some negative experience over the course of treatment, consistent with the transient distress that accompanies exposure-based treatment and did not differ between AI and human feedback. Taken together, the findings suggest that psychologist-reviewed AI-generated feedback can support meaningful improvement in social anxiety at a level comparable to human feedback and with no detectable difference in reported negative effects, and that the principal barrier to AI-supported ICBT may lie in expectations rather than in outcomes.

Several caveats bound these conclusions. First, the AI condition estimated the effect of AI-drafted feedback that was reviewed by a licensed psychologist before delivery; generalization to fully autonomous, unvetted AI feedback therefore extends beyond the present design (Stade et al., 2024). Second, human feedback was provided by several therapists whose messages were pooled into a single condition; therapist-level clustering in the rate of change was not modeled, as the therapist-level slope variance could not be reliably estimated at the realized sample (Magnusson et al., 2018). Third, post-treatment data were available for roughly half of allocated participants, and the intention-to-treat estimates accordingly rest on the assumption that missingness depended only on observed information; although likelihood-based mixed models are robust to missingness of this kind, departures from this assumption cannot be ruled out (Bell et al., 2013). Fourth, recruitment that mentioned AI may have selected participants more willing to receive AI-generated feedback, which limits generalizability to people who would decline such treatment. Fifth, the pooled comparison of all four feedback arms with unguided self-help did not detect a benefit of feedback, which leaves assay sensitivity for the source comparison uncertain. The value of support may depend on treatment and delivery context, and these findings do not establish that support is dispensable. Finally, pre-treatment credibility was rated under an imagination instruction, and the observed expectation–outcome dissociation presumes that imagined credibility corresponds to the credibility that operates during actual treatment. Within these bounds, the pattern of results is consistent, with comparable outcomes across feedback sources despite markedly asymmetric prior expectations.

## Ethical statement

This study has been reviewed and approved by the Swedish Ethical Review Authority on 17 March 2025 (Dnr: 2025–01058-01).

## Declaration of Generative AI and AI-assisted technologies in the writing process

During the preparation of this work the authors used Claude (Anthropic) in order to assist with language editing. After using this tool, the authors reviewed and edited the content as needed and take full responsibility for the content of the published article.

## Funding

This research did not receive any specific grant from funding agencies in the public, commercial, or not-for-profit sectors.

## Declaration of competing interest

Per Carlbring and Gerhard Andersson are editors of Internet Interventions. Neither had any involvement in the peer review of this article, and neither has access to information about its peer review. Full responsibility for the editorial process for this article was delegated to another journal editor. Per Carlbring has received honoraria from OpenAI within the past three years. The authors declare no other competing interests.

## Data Availability

The data that support the findings of this study are available from the corresponding author, PC, upon reasonable request.
