# Supplementary material for: AI-generated vs. human therapist feedback in internet-based CBT for social anxiety disorder: A six-arm randomized controlled trial with deception design
Source: Internet Interv. 2026 Oct 16;46:101012. doi: 10.1016/j.invent.2026.101012 (PMC13698080; doi:10.1016/j.invent.2026.101012)
Supplement: Supplementary file 1 [file mmc1.docx]

**Supplementary Material**

**Figure S1.**

Assumed data-generating process underlying the primary analyses. Randomization renders both exposures (actual and believed feedback source) exogenous, so the total-effect contrasts require no covariate adjustment (empty adjustment set). Belief about the feedback source is assumed to mediate the effect of the told-source manipulation; feedback content mediates the effect of actual source. Baseline credibility/expectancy (CEQ) is a measured common cause of alliance and outcome. Dropout is a missingness indicator: the intention-to-treat mixed models assume missingness depends only on observed variables (MAR), whereas completer analyses condition on Dropout and are therefore vulnerable to selection effects if post-treatment symptom level influenced dropout (dashed assumption, see Limitations).

**Table S1.**

*Treatment modules and homework.*

| **Module** | **Theme** | **Main content** | **Homework and practice described in the manual** |
| --- | --- | --- | --- |
| 1 | Understanding social anxiety | Symptoms, avoidance, and the rationale for CBT | Describe personal symptoms and their impact; identify feared situations and construct an initial anxiety hierarchy. |
| 2 | The role of thoughts | Automatic thoughts, underlying assumptions, and the cognitive model of social anxiety | Record negative automatic thoughts and describe a personal maintenance cycle. |
| 3 | Thinking traps and challenging thoughts | Cognitive distortions, alternative interpretations, and treatment goals | Record and challenge negative thoughts; identify thinking traps and set treatment goals. |
| 4 | Testing thoughts through behavior | Behavioral experiments and difficulties in challenging thoughts | Plan and conduct behavioral experiments; record predictions, outcomes, and alternative interpretations. |
| 5 | Exposure | Graded exposure to feared social situations | Develop detailed anxiety hierarchies; plan, conduct, and record exposure exercises. |
| 6 | Attention and focus | Self-focused attention, shifting attention, and safety behaviors | Practice shifting attention and continue exposure; compare social situations with and without safety behaviors. |
| 7 | Continued exposure | Troubleshooting exposure and confronting feared situations | Identify safety behaviors and change them during continued exposure. |
| 8 | Social skills | Listening, conversation, nonverbal communication, and assertiveness | Practice communication skills, reflect on assertiveness, and continue exposure and thought records. |
| 9 | Consolidation and relapse prevention | Perfectionism, self-confidence, setbacks, and maintenance | Prepare a personal action plan, review treatment goals, and plan further practice. |

*Note.* Homework summarizes practice described in the treatment manual (Furmark et al., 2019). Modules were released weekly. Participants worked through them at their own pace and submitted written homework through the platform.

**Table S2**

*Recorded message use and module engagement by treatment arm.*

| **Actual source / believed source** | **n** | **Feedback-related messages read, M (median)** | **Participant-sent messages, M (median)** | **Modules opened, M (median)** | **Modules completed, M (median)** |
| --- | --- | --- | --- | --- | --- |
| AI / AI | 44 | 4.1 (3.5) | 4.5 (1.0) | 4.9 (4.5) | 4.0 (3.0) |
| Human / human | 45 | 3.2 (3.0) | 3.2 (2.0) | 4.8 (4.0) | 4.0 (3.0) |
| AI / human | 44 | 4.5 (5.0) | 6.0 (2.0) | 5.7 (6.0) | 4.5 (5.0) |
| Human / AI | 43 | 3.7 (2.0) | 3.2 (2.0) | 5.8 (6.0) | 4.8 (5.0) |
| Self-guided | 44 | 0.0 (0.0) | 0.3 (0.0) | 4.6 (4.5) | 3.5 (3.0) |

*Note.* Message counts cover the first 63 days from treatment start and include participants without recorded message activity as zero. Feedback-related messages were identified by subject headings, including feedback, progress enquiries, welcome messages, and closing messages; repeat reads were deduplicated. Unread messages were not captured. Participant-sent messages are recorded writes and replies. Module counts come from the platform statistics export and are not restricted to the 63-day window; completion denotes full recorded progress through a module. Pooled by actual source (88 participants per source), mean feedback-related messages read were 4.3 (SD = 3.0; median = 4.0) for AI-generated and 3.4 (SD = 2.7; median = 2.5) for human therapist feedback, and mean participant-sent messages were 5.3 (SD = 12.0; median = 2.0) and 3.2 (SD = 3.2; median = 2.0). These counts do not measure message length or clinician time.
